# Histopathological Features and Clinical Findings of Feline Chronic Gingivostomatitis (FCGS) in Cats (*Felis catus*) at Gloriavet Clinic, Bandung

**DOI:** 10.1155/vmi/2438106

**Published:** 2026-08-21

**Authors:** Febriani Rohmatul Azizah, Grace Julianty Hutomo, Chrysanti Murad, Shafia Khairani

**Affiliations:** ^1^ Study Program of Veterinary Medicine, Padjadjaran University, Jatinangor 45363, Indonesia, unpad.ac.id; ^2^ Gloriavet Clinic, Bandung 40163, Indonesia; ^3^ Department of Biomedical Science, Padjadjaran University, Jatinangor 45363, Indonesia, unpad.ac.id

**Keywords:** clinical findings, feline chronic gingivostomatitis (FCGS), histopathology, immune responses, Stomatitis Disease Activity Index (SDAI)

## Abstract

Feline chronic gingivostomatitis (FCGS) is a chronic inflammatory condition of the oral cavity characterized by distinctive lesions involving various areas of the mucosa. Histopathological examination was performed to differentiate FCGS from other inflammatory conditions and to assess its severity for diagnostic purposes. The aim of this study was to evaluate the clinical findings and histopathological features of oral lesions in cats diagnosed with FCGS and to examine their association with patient‐related factors, including breed, sex, age, and housing conditions. A semiquantitative approach was used to assess clinical findings using the Stomatitis Disease Activity Index (SDAI) and histopathological lesion scoring. The results revealed pathological findings such as erythema, swelling, hemorrhage, and ulceration, with the most severe lesions commonly observed in the gingival region associated with periodontal problems. Histopathological findings included epithelial hyperplasia, intraepithelial inflammation cell infiltration, spongiosis, and ulceration as indicators of epithelial damage, along with inflammatory cell infiltration within the lamina propria and submucosa, with varying degrees of severity. Statistical analysis showed that patient‐related variables had no significant influence on the severity of either clinical or histopathological findings (*p* > 0.05). These findings suggest that the progression and severity of FCGS are closely associated with immune responses to multiple pathogens within the oral mucosa rather than by individual patient factors.

## 1. Introduction

The oral cavity supports various essential functions in the daily lives of animals. Disorders affecting this region can significantly impact overall health and quality of life (QoL) [1, 2]. Feline chronic gingivostomatitis (FCGS) is a common pathological condition observed in small animal clinical practice, with a reported prevalence of 26.6% in a study of feral cats in South Korea [3], and 0.7% at veterinary clinics in North West England [4]. This condition is characterized by chronic inflammation involving multiple regions of the oral mucosa, including the gingiva, tongue, palate, and pharynx [5]. Clinically, manifestations of FCGS are halitosis, hypersalivation, anorexia, dysphagia, gingival bleeding, and weight loss [6]. The severe pain associated with this condition can markedly reduce QoL and, in advanced cases, may pose a life‐threatening risk to affected cats [7, 8].

FCGS is a multifactorial disease primarily involving immune responses to infectious agents and alterations in the oral microbiome [9]. Viral pathogens implicated in its pathogenesis include feline calicivirus (FCV), feline herpesvirus (FHV‐1), feline leukemia virus (FeLV), and feline immunodeficiency virus (FIV), which are associated with chronic and latent infections, particularly within the oropharyngeal region. In addition, bacteria such as *Pasteurella multocida* and *Bartonella* spp. may contribute to inflammation, especially in the presence of dysbiosis and periodontal colonization [7, 10]. Several nonpathogenic risk factors have also been reported. Purebred cats, such as Persians and Siamese, appear more frequently affected and are considered to have a breed predisposition. Adult cats aged 7–10 years are also more susceptible, likely due to age‐related shifts in the oral microbiome. Male cats with outdoor access are similarly at higher risk due to increased environmental exposure to infectious agents. However, the relationship between these factors and the underlying mechanisms of FCGS remains unclear, and further investigation is necessary [11–14].

Histopathological examination of oral lesions plays an important role in assessing the severity of inflammation, differentiating FCGS from other oral pathological conditions, and determining prognosis. Histopathological features that have been constantly reported in previous studies include lymphoplasmacytic infiltration, epithelial ulceration, and degenerative epithelial changes indicating chronic inflammation [15–17]. These tissue alterations form the basis of histopathological scoring for FCGS, categorized on a scale from 0 to 3 [18]. According to one study, 84.6% of FCGS cases fall within Grade 3, indicating severe inflammatory changes [19].

Histopathological evaluation of oral lesions in FCGS is therefore critical for determining the underlying etiology of the disease [17]. This study aims to describe the histopathological characteristics of oral lesions in cats diagnosed with FCGS and to identify associations between breed, sex, age, and living environment with the severity of clinical and histopathological findings. The results of this research are expected to provide a comprehensive overview of the characteristics and developmental patterns of FCGS.

## 2. Materials and Methods

This study employed a cross‐sectional design with a population consisting of cats (*Felis catus*) diagnosed with FCGS who underwent dental extraction at Gloriavet Clinic, Bandung. The sample size was determined by case availability during the data collection period (July–September 2025), which resulted in 12 samples of oral lesions.

### 2.1. Sample Collection

Medical record data, including breed, sex, age, housing environment, clinical findings, and results of some diagnostic tests such as complete blood count (CBC), blood biochemistry, and polymerase chain reaction (PCR), were collected and served as supporting data in the analysis.

Clinical signs were assessed using the Stomatitis Disease Activity Index (SDAI), a scoring system used to evaluate the severity of stomatitis lesions, determine prognosis, and monitor disease progression or therapeutic response during follow‐up examinations [8, 20]. Owner evaluation and veterinarian assessment of body weight and oral lesions in regions are included in this scoring (Table 1).

**TABLE 1:** Stomatitis Disease Activity Index (SDAI) used in this study.

**Score**	**Description**			
	**Owner evaluation**	**Weight evaluation**	**Inflammation evaluation**	

0	• Appetite: eating normally	Gain ≥ 0.5 kg	None	
	• Activity level: normal activity level			
	• Grooming: grooming normally			
	• Perceived comfort: normal			
1	• Appetite: eating wet and dry food, but less than normal amount	Gain ≥ 0.25 kg, but < 0.5 kg	Mild	
	• Activity level: plays spontaneously, but not frequently			
	• Grooming: grooming excessively			
	• Perceived comfort: uncomfortable			
2	• Appetite: eats only wet food on his/her own	Gain < 0.25 kg	Moderate hemorrhage may occur when the lesion is gently touched (for example, with a cotton bud)	
	• Activity level: low activity level, but will play occasionally			
	• Grooming: grooms occasionally			
	• Perceived comfort: uncomfortable			
3	• Appetite: eats only pureed food, or only when hand fed	Weight loss	Severe, spontaneous hemorrhage	
	• Activity level: no interest in people or other pets, spends most of time sleeping			
	• Grooming: will not groom			
	• Perceived comfort: extremely painful			

**Stomatitis Disease Activity Index**	**0**	**1**	**2**	**3**

Owner evaluation (average)				
Weight evaluation				
Maxillary buccal mucosal inflammation				
Mandibular buccal mucosal inflammation				
Maxillary attached gingival inflammation				
Mandibular attached gingival inflammation				
Palatoglossal folds inflammation				
Molar salivary gland inflammation				
Oropharyngeal inflammation				
Lingual and/or sublingual inflammation				
Total (maximal = 30)				

Oral lesion samples in this study were obtained through incisional biopsy, particularly around the gingiva region, as this site provided the most practical and accessible location for tissue collection during the extraction process. Both SDAI scoring and sample collection were performed once, prior to the initiation of dental extraction.

### 2.2. Histopathological Examination

All samples were processed into histopathological slides using standardized histotechnical procedures and stained with hematoxylin–eosin (H&E) prior to analysis. Three samples were excluded from histopathological evaluation due to unreadable tissue quality, although their clinical findings were still included in the discussion.

Slides were examined under an Olympus CX33 microscope equipped with an EP50 camera at 40x, 100x, and 400x magnification. Histopathological assessment of oral lesions in FCGS cases was performed according to the criteria summarized in Table 2 [18].

**TABLE 2:** Histopathological scoring used in this study.

Grade	Description	Features
0	Normal. (baseline) •Stratified squamous epithelium with sparse intraepithelial lymphocytes •The lamina propria/submucosa contains sparse scattered mast cells and lymphocytes. The lymphocytes may form small subepithelial aggregates	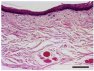

1	Mild •Variable, mild epithelial hyperplasia and parakeratosis. May have slightly increased numbers of intraepithelial lymphocytes and sparse exocytotic neutrophils •The lamina propria/submucosa contains a sparse to light, perivascular to interstitial population of plasma cells, lymphocytes, mast cells, and rare macrophages	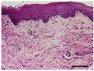

2	Moderate •Epithelial hyperplasia with some regions of degeneration or ulceration. Mild to moderate numbers of intraepithelial lymphocytes often mixed with macrophages and neutrophils •The lamina propria/submucosa contains a moderate inflammatory cell infiltration of lymphocytes and plasma cells mixed with variable numbers of macrophages and neutrophils. The inflammatory cells may form a distinct “lichenoid” band within the superficial lamina propria. In the submucosa, the infiltrating cells often extend around skeletal muscle	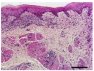

3	Severe •Often extensive regions of epithelial degeneration, spongiosis, and ulceration and superficial exudation with many macrophages, neutrophils, and lymphocytes •The lamina propria/submucosa contains a dense inflammatory infiltrate with variable proportions of lymphocytes, plasma cells, macrophages, and neutrophils. In some sections, the lamina propria is expanded or replaced by immature granulation tissue and fibrin necrotic debris	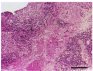

### 2.3. Statistical Analysis

Data were organized in Microsoft Excel. Ordinal variables, including age, SDAI scores, and histopathology scores, were tested for normality using the Shapiro–Wilk test. Associations between ordinal independent variables and ordinal dependent variables were analyzed using Spearman’s rho, while associations involving nominal independent variables and ordinal outcomes were assessed using the Kruskal–Wallis test. Statistical analyses were performed using SPSS.

## 3. Results and Discussion

### 3.1. Clinical Findings of FCGS

A total of 12 cats were included in the study during the data collection period (Table 3). Most patients showed SDAI scores within the moderate inflammation category, with a mean score of 17.75, indicating intermediate clinical severity with lesions localized to specific oral regions. SDAI assessment was performed only once prior to dental extraction, which all patients had already received preextraction therapy. Delays before extraction occurred due to the clinic’s surgical reservation system and also, in several cases, due to unstable preoperative hematology or biochemistry results, such as anemia or metabolic disturbances, which required stabilization to reduce anesthetic and surgical risks.

**TABLE 3:** Data results of patient‐related variables, SDAI, and histopathological score.

Sample name	Breed	Sex	Age	Housing type	SDAI score	Histopathological score
A1	Mix	*M* (*n*)	3	Indoor	17	—
A2	Dom	*M* (*n*)	3	Indoor	15	1
A3	Dom	*M* (*n*)	3	Indoor	14	2
A4	Dom	*F*	2	Indoor	15	2
A5	Mix	*F* (*s*)	3	Indoor	13	2
A6	Dom	*F* (*s*)	3	Indoor	19	—
A7	Persia	*F* (*s*)	3	Indoor	22	3
A8	Persia	*M* (*n*)	3	Indoor	21	2
A9	Dom	*F* (*s*)	3	Indoor	17	—
A10	Persia	*F*	3	Indoor	9	2
A11	Persia	*M* (*n*)	3	Indoor	27	3
A13	Dom	*F* (*s*)	4	Indoor	24	2

*Note:* Dom = domestic; Mix = mix‐domestic. Age: 2 = junior (6 months–2 years); 3 = adult (2–10 years); 4 = senior (> 10 years).

Abbreviations: *F* = female, *F*(s) = female spayed, *M* (*n*) = male neutered.

Preextraction therapy included anti‐inflammatory, antibiotics, and vitamin or immunomodulatory supplementation. NSAIDs such as meloxicam were used for pain and inflammation control, while antibiotics including long‐acting amoxicillin and clindamycin helped manage opportunistic periodontal bacteria and prevent systemic infection [21, 22]. Supplementation, typically vitamin C or immune modulators such as 4Life Transfer Factor Tri‐Factor, supported tissue repair and immune function [23, 24]. Collectively, these treatments helped reduce active inflammation, promote tissue healing, and modulate mucosal immune responses to chronic infection [25].

Considering this therapeutic intervention administered, the variability in oral lesion severity may have been influenced by differences in treatment type, duration, and individual patient response. This factor is also relevant when interpreting histopathological findings, since biopsy samples in this research were also collected only once at the time of extraction. Thus, the severity of both clinical and histopathological lesions may represent not only the underlying disease processes but also potential reductions in inflammation resulting from previous treatments.

Medical records indicated that several FCGS patients in this research responded positively to preoperative treatment, as shown by reduced mucosal inflammation and improvements in appetite and body weight compared to previous examinations. However, previous studies have reported that treatments such as anti‐inflammatory agents, antibiotics, and analgesics generally provide only temporary relief of clinical signs in cats with stomatitis [26]. Consistent with these findings, several patients in this study had experienced recurrent inflammation for months to years, showing short‐term improvement with prior medications, before eventually requiring dental extraction. Thus, beyond controlling primary pathogens, tooth extraction is considered as the definitive therapy for reducing inflammation in FCGS. Extraction focuses on managing periodontal tissues to eliminate the oral microbiota associated with disease progression [27].

Recent data indicate that 32.4% of feline tooth extractions are attributed to FCGS, with 65.51% of these involving a combination of FCGS and periodontitis, followed by FCGS alone, and cases involving FCGS, periodontitis, and tooth resorption. These findings show the relation between oral inflammation and dental pathology and highlight the importance of dental hygiene, routine examinations, timely diagnosis, and appropriate intervention [28].

Owner‐reported evaluations often included easily observable clinical signs such as hypersalivation with halitosis, altered appetite, and pain when opening the mouth. Changes in daily activity or grooming were less commonly noted and typically manifested as poor coat condition. Previous studies have shown that these signs reflect oral discomfort associated with inflammatory pain [29]. Although saliva production serves as a physiological defense mechanism, painful oral conditions such as FCGS may impair swallowing, resulting in saliva retention. Consequently, hypersalivation may reflect not only an increase in salivary secretion but also the presence of dysphagia secondary to active inflammation, making the accumulation of saliva more pronounced [30].

**FIGURE 1:**
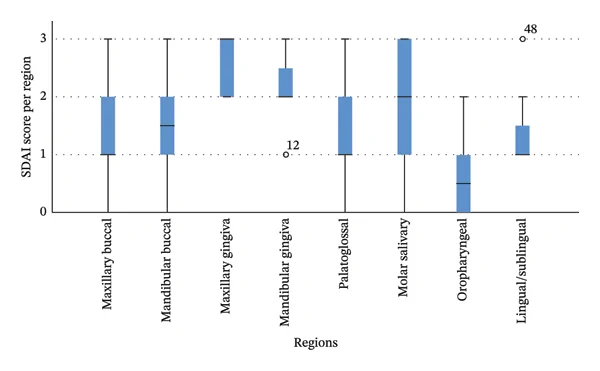
Boxplot analysis for distribution of severity in regions observed with SDAI.

Most cases showed inflammation characterized by mucosal erythema, swelling, and hemorrhage. Variability in the severity and distribution of lesions was observed among individuals, with some showing diffuse inflammation and others localized involvement. The distribution of inflammatory severity across mucosal regions based on SDAI scoring is presented in Figure 1.

**FIGURE 2:**
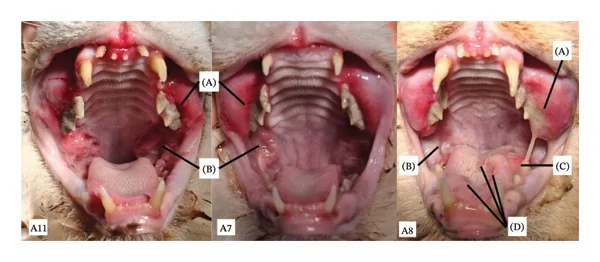
Variation in the severity of lesions across the examined regions. (A) Attached gingiva. (B) Buccal mucosa. (C) Molar salivary gland. (D) Lingual. All three patients exhibited severe inflammation in the gingival region. In the buccal to caudal mucosa, the severity varied, (A11) showing severe inflammation, (A7) mild inflammation, and (A8) no inflammation, and (A8) also demonstrated inflammation in the molar salivary gland and ulceration on the lingual surface.

The highest lesion scores, based on the boxplot analysis, were predominantly observed in the maxillary attached gingiva, indicating a relatively greater degree of inflammation in this region (Figure 2). Periodontal disease affecting the premolar and molar teeth appears to be closely associated with the severity of inflammation in the attached gingiva, where lesions tend to persist more than in the other mucosal regions. This condition is primarily triggered by plaque accumulation as a bacterial biofilm on tooth surfaces that may calcify into tartar (calculus) [31]. Plaque contains various pathogenic bacteria, including *Porphyromonas gingivalis*, which is one of the bacteria that is frequently identified in feline periodontal disease. As plaque spreads, it can invade adjacent tissues and stimulate host immune responses, contributing to the development of gingivitis and periodontitis [32].

Previous studies have reported that periodontal lesions may progress into FCGS, influenced by individual immune responses to inflammation initially triggered by plaque deposition, as well as genetic, environmental, and viral infection [33] The chronicity of FCGS is also linked to alterations in the oral microbiome, with affected cats exhibiting increased abundance of *Mycoplasma felis* and fungi such as *Aspergillus* and *Candida*, which can prolong inflammatory processes [9].

PCR testing of FCGS patients in this study further detected viral agents known to contribute to disease pathogenesis. Among the 12 patients, six tested with PCR: four cats showed single infections with FCV, while two exhibited co‐infection with FCV and FHV. FCV is one of the common respiratory pathogens that can also induce oral lesions due to viral shedding through saliva [34]. Ulcerative lesions on the lingual surface are a well‐documented presentation of FCV infection, and their severity does not appear to correlate with viral load [10]. This aligns with findings from this study, which also identified similar ulcerative lesions in FCV‐positive cats (Figure 2 A8). FHV is likewise associated with FCGS through its ability to modulate host immune responses and exacerbate inflammation [13]. FHV replication within the oral mucosa may increase salivary secretion and, in some cases, lead to ulcerative lesions similar to those caused by FCV, due to its cytolytic effects on epithelial cells [35].

### 3.2. Histopathological Features of FCGS

Biopsy samples were obtained from all patients included in the clinical assessment. Three slides exhibited suboptimal quality, preventing microscopic evaluation and histopathological scoring. This limitation was likely influenced by extrinsic factors during tissue collection, processing, or slide preparation and highlights the need for procedural refinement in future studies.

Microscopically, tissue alterations and the degree of mucosal damage were evaluated across three regions, the epithelium, lamina propria, and submucosa, to facilitate final severity assessment. Most FCGS cases in this study were classified as having moderate inflammation, with a histopathology score of 2.

**FIGURE 3:**
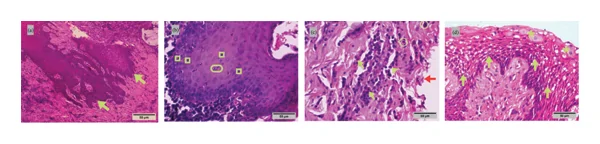
Variation of histopathological findings occur in the epithelial layer (a). Epithelial hyperplasia, noted by basal cell proliferation and elongation of rete pegs (arrow) (H&E, 100x). (b) Intraepithelial inflammatory cell infiltration within hyperplasia epithelium, lymphocyte (square), and neutrophil (ellipse). (c) Ulceration, shown by exposed lamina propria (red arrow), with inflammatory cell infiltration within lamina propria including macrophage (round), plasma cell (arrowhead), and Mott cell (arrow). (d) Spongiotic epithelium (arrow) with superficial exudation (asterisk) (H&E, 400x).

A variety of epithelial lesions were identified, including hyperplasia, intraepithelial inflammatory cell infiltration, ulceration, and spongiosis (Figure 3). These findings varied not only between samples but also within different microscopic fields of the same slide, indicating heterogeneous degrees of epithelial damage. This variation may be influenced by differences in epithelial degenerative or regenerative activity, which follows a sequential process involving expansion of healthy cells, dedifferentiation, migration, proliferation, and redifferentiation. Mechanical forces applied to specific mucosal surfaces may also contribute to these regenerative patterns [36, 37].

The epithelium serves as a protective barrier against irritants, mechanical trauma, and microbial invasion. Epithelial lesions such as hyperplasia, reflecting basal cell proliferation as a protective response, were prominent in this study. Eight of nine samples showed hyperplasia, characterized by increased epithelial cell numbers, epithelial thickening, and elongation of rete pegs extending into the lamina propria. Hyperplasia is often associated with inflammatory cell infiltration in the lamina propria. Similar patterns have been described in human pathology under the term pseudoepitheliomatous hyperplasia, a reactive proliferation resulting from infection, inflammation, or degeneration, and distinguishable from neoplastic hyperplasia seen in squamous cell carcinoma [38, 39].

Another commonly observed epithelial change was increased intraepithelial inflammatory cell infiltration, consisting of both lymphocytes and polymorphonuclear cells, particularly neutrophils. All samples that exhibited hyperplasia in this study also demonstrated intraepithelial infiltrates with varying density. This finding is consistent with previous reports describing neutrophil aggregates within hyperplastic epithelium [40]. Intraepithelial lymphocytes contribute to epithelial injury, especially at the basement membrane, through cytotoxic mechanisms similar to those described in oral lichen planus, squamous cell carcinoma, and microbial infections associated with periodontitis [41]. Meanwhile, neutrophil infiltration can occur in certain epithelial types such as the gingival junctional epithelium, which contains wider intercellular spaces that facilitate cell migration from the lamina propria. Increased neutrophil presence generally represents a response to bacterial infection and is driven by chemotactic cytokines produced by epithelial cells following exposure to bacterial lipopolysaccharides in periodontal tissues [42, 43].

An increase in intraepithelial inflammatory cell infiltration together with epithelial hyperplasia generally reflects mild to moderate lesions. However, in more advanced conditions, chronic inflammatory processes may trigger extensive epithelial damage, including the development of ulcerative lesions. These lesions are typically characterized by the loss of epithelial structure, exposing the basement membrane and the underlying connective tissue. In some cases, the lesions may extend into deeper structures such as fascia, muscle, or even bone [7].

Such ulcerative changes may be associated with a range of etiologies, including bacterial, viral, or fungal infections, and immune‐mediated reactions, trauma, and neoplastic processes [44].

Unlike ulceration, which represents complete loss of the epithelial layer, spongiosis reflects a distinct epithelial lesion associated with severe inflammation and more heterogeneous tissue damage. Histopathological examination showed intercellular edema within the epithelium. Similar lesions have been reported in cases of canine chronic ulcerative stomatitis (CCUS), where spongiosis is considered indicative of epithelial degeneration [45]. However, the underlying mechanisms remain incompletely understood. In human studies, spongiotic hyperplasia has been associated with several contributing factors, including plaque and calculus accumulation, trauma, viral infections, and hormonal influences [46].

**FIGURE 4:**
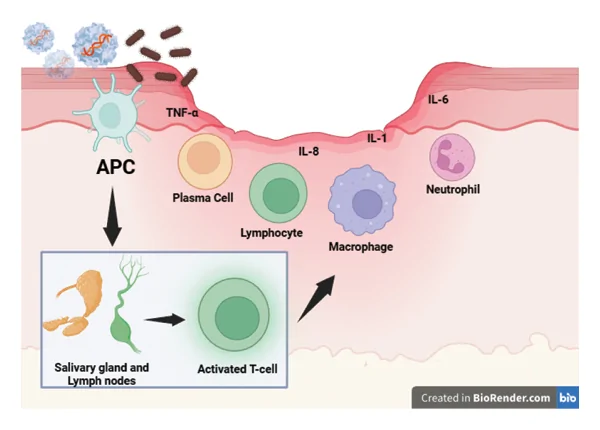
Cellular mechanism of immune response in epithelial damage induced by pathogens in epithelial surface (personal illustration, made in Biorender.com).

Epithelial damage is driven by interactions between invading pathogens and the host immune response within the mucosal layer. Antigens are captured by antigen‐presenting cells (APCs) in the epithelium and transported to lymphoid tissues such as lymph nodes, tonsils, and salivary glands. These APCs activate T cells, including *T* helper cells—which regulate other immune effectors such as macrophages, plasma cells, and neutrophils at the infection site—and cytotoxic T cells, which directly induce apoptosis of pathogen‐infected epithelial cells [16]. Sustained antigenic stimulation promotes cytokine production, leading to disruption of intercellular junctions and ultimately contributing to epithelial ulcer formation [47] (Figure 4).

**FIGURE 5:**
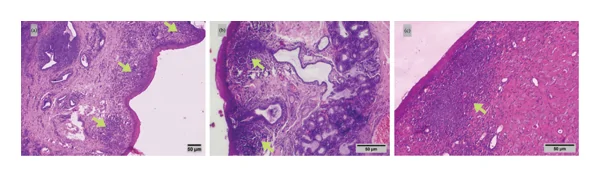
Lichenoid pattern, noted by inflammatory cell infiltration within the lamina propria, extending to the basal membrane of the epithelium. (a) Low‐magnification view (H&E, 40×) showing the overall lichenoid inflammatory pattern. (b) Higher‐magnification view (H&E, 100×) demonstrating inflammatory cell infiltration within the lamina propria extending toward the basal membrane. (c) Higher‐magnification view (H&E, 100×) highlighting the inflammatory infiltrate adjacent to the basal layer of the epithelium.

The epithelial alterations observed in this study are closely associated with infiltration of inflammatory cells within the lamina propria. In several cases, these cells accumulated multifocally in the superficial (papillary) lamina propria extending to the epithelial basement membrane, forming a lichenoid pattern (Figure 5). This pattern represents T‐cell–mediated inflammation and is often accompanied by basal cell degeneration, which may progress to ulceration in more severe cases [44, 48].

Lichenoid in FCGS closely resembles oral lichen planus in humans, a condition driven by cell‐mediated hypersensitivity and considered a chronic autoimmune reaction to epithelial antigens, including microbes, with stress acting as an exacerbating factor [49]. This etiology aligns with previous FCGS studies suggesting a multifactorial process, in which immune responses to infectious agents and environmental stressors play central roles in disease progression [50]. These immunopathological similarities have also been used as a comparative reference in recent FCGS research [15].

The initial development of lichenoid inflammation in lichen planus is triggered by cellular mechanisms similar to those observed in the epithelium of FCGS lesions. Antigen exposure leads to lymphocyte activation, particularly cytotoxic T cells, which release molecules such as perforin that induce epithelial apoptosis. This supports the presence of a lichenoid pattern within the lamina propria, inducing epithelial damage [51].

**FIGURE 6:**
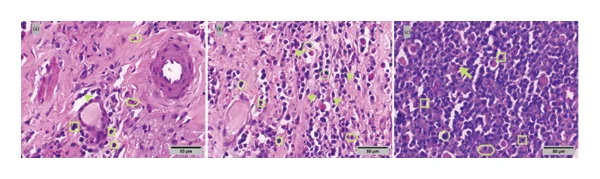
Variation of inflammatory cell infiltration density within submucosa. (a) Mild. (b) Moderate. (c) Severe. Lymphocyte (square), neutrophil (ellipse), macrophage (round), plasma cell (arrowhead), and Mott cell (arrow) (H&E, 400x).

In the submucosa, the primary parameter assessed was the density of inflammatory infiltrates. This layer contains structures such as salivary glands, blood vessels, and smooth muscle, where inflammatory cells commonly accumulate. The degree of infiltration varied within individual samples across different microscopic fields, and focal inflammation was also observed (Figure 6). Consequently, the severity of lesions within a single sample ranged across categories, from mild to moderate or moderate to severe.

Inflammation is an essential component of the immune response, serving to eliminate invading pathogens and initiate tissue repair. It becomes chronic when it persists for months or years, typically due to pathogens that cannot be fully cleared, resulting in sustained production of proinflammatory cytokines and prolonged immune‐cell activity [52, 53].

Viral infections such as FCV can maintain chronic inflammation throughout the course of FCGS, as the virus may persist or cause recurrent infections in susceptible individuals. High genetic variability among FCV strains often leads to inconsistent PCR results across examinations [54, 55]. In addition, several studies have demonstrated a strong link between FCGS and the presence of pathogenic bacteria in periodontal tissues. Oral pathogens manipulate the innate immune response through immune‐evasion mechanisms, enabling long‐term infection and persistent inflammation. These bacteria stimulate the release of cytokines and chemokines—including IL‐1, IL‐6, IL‐8, and TNF‐*α*—which contribute to epithelial damage and upregulation of immune receptors. Persistent co‐infection with multiple pathogens promotes prolonged inflammation and facilitates lesion spread across different mucosal regions, producing the characteristic inflammatory patterns seen in clinical FCGS [56, 57].

Histopathological lesions in FCGS typically contain lymphocytes, plasma cells, macrophages, and neutrophils. Lymphocyte and plasma‐cell infiltration indicates an adaptive immune response both locally and systemically. Plasma cells and macrophages also express toll‐like receptors (TLRs), enabling recognition of pathogen‐associated molecular patterns (PAMPs) from bacteria, viruses, and fungi, contributing to disease pathogenesis [15]. Neutrophil recruitment, commonly observed in chronic periodontitis, promotes the release of antimicrobial proteins and reactive oxygen species (ROS), which exacerbate tissue damage and support the persistence of chronic lesions [58].

A notable histopathological feature in FCGS is the presence of Mott cells. These cells represent a differentiated form of plasma cells containing Russell bodies, which are accumulations of immunoglobulins produced during late‐stage plasma‐cell activity. Mott cells commonly appear in tissues with dense plasma‐cell infiltration and are considered indicators of persistent chronic immune activation [59, 60].

The histopathological lesions observed in this study indicate that mucosal inflammation in FCGS patients persists despite the administration of preextraction treatments. These findings strengthen existing evidence that the inflammatory process is driven by multiple pathogens and remains persistent, as demonstrated by the continued presence of chronic inflammatory cell infiltrates even after attempts to control triggering factors, such as bacterial load and active inflammation through antibiotic and anti‐inflammatory therapy. Although this study employed a cross‐sectional design and therefore could not evaluate clinical outcomes following dental extraction performed after SDAI assessment and biopsy collection, the results align with previous reports showing that medical therapy alone is insufficient to resolve the ongoing inflammatory process in FCGS.

### 3.3. Association of Breed, Sex, Age, and Housing Environment With the Severity of Clinical Findings and Histopathological Lesions

Statistical analysis was performed on only nine samples with evaluable histological preparations. Analysis results showed that none of the independent variables was significantly associated with the severity of FCGS, either clinical or histopathological (*p* > 0.05). Nevertheless, several variables, particularly breed, age, and housing environment, displayed patterns consistent with previous studies, suggesting that patient background may influence the incidence of FCGS [12]. In contrast, sex was not associated with disease occurrence in this study. The compilation of patient data, including SDAI assessment and histopathological scoring, is summarized in Table 3.

Purebred cats, such as Persians, are known to have a higher predisposition for periodontal disease, likely due to brachycephalic craniofacial morphology that results in crowded or malpositioned teeth [61]. This predisposes affected teeth to excessive mechanical stress and suboptimal mastication, increasing the risk of periodontal pathology. Similar findings have been associated with inflammatory tooth resorption, particularly in premolars [62], which may help explain the more severe inflammatory lesions observed in the maxillary attached gingiva in this study.

Recent literature provides a different perspective, reporting higher FCGS prevalence among domestic shorthair cats compared to purebreds, likely due to incomplete vaccination histories and earlier exposure to infectious agents in outdoor environments [28]. This may explain why FCGS cases in this study were distributed relatively evenly across domestic, mixed‐breed, and Persian cats. Although purebreds are clinically more prone to periodontal disease, breed was not statistically associated with disease severity in the present study.

Regarding housing conditions, all cats included in this study were kept indoors at the time of examination; thus, environmental variation was insufficient to assess its influence on disease severity. However, based on owner information, several cats were rescued from outdoor settings, suggesting that the pathological process may have begun prior to adoption, possibly driven by persistent viral infections given the chronic and progressive nature of FCGS.

Four cats in this study also came from the same household, suggesting possible shared exposure to infectious agents. This is consistent with prior studies reporting that FCV spreads efficiently in multicat environments and that FCGS is more common in multi‐cat households, where viral exposure and environmental stress are increased [63, 64].

Both male and female cats were affected, and sex was not associated with disease severity, aligning with previous findings [11]. Although some studies have suggested higher risk in males, this association is considered speculative and may be confounded by environmental factors, as males are more likely to have outdoor access and thus higher exposure to infectious agents [12].

Most cats in this study were adults aged 2–10 years. Although age was not statistically associated with disease severity, this distribution aligns with previous reports describing FCGS as most common in adult cats [11]. Age may still influence inflammatory severity due to immunosenescence, which affects oral mucosal immunity and increases risk for periodontal disease, particularly in cats over 10 years old, although not always accompanied by FCGS [28].

Overall, the findings of this study, together with previous literature, indicate that individual factors such as breed, age, and housing background may be associated with FCGS incidence but generally do not correlate with disease severity except for age, which may influence susceptibility to infection and inflammation. These patterns suggest that the progression and severity of FCGS are driven primarily by interactions between pathogens and the host immune response within the oral mucosa, which collectively sustain and amplify chronic inflammation, as reflected in both clinical and histopathological findings.

## 4. Conclusions

The histopathological features of oral lesions in cats with FCGS at Gloriavet Clinic, Bandung, demonstrated epithelial hyperplasia, ulceration, spongiosis, and chronic inflammatory cell infiltration including lymphocytes, plasma cells, macrophages, and Mott cells across the epithelium, lamina propria, and submucosa. Statistical analysis showed that breed, sex, age, and housing environment were not significantly associated with the severity of clinical findings assessed using the SDAI, nor with the degree of histopathological lesion severity.

### 4.1. Research Limitation

Variability in the degree of inflammation observed in both clinical findings and histopathological lesions may reflect heterogeneity in individual responses to therapies administered prior to SDAI assessment and biopsy collection. The relatively small sample size and uneven data distribution also limit the strength and generalizability of the conclusions, highlighting the need for future studies with larger and more diverse populations. In addition, because biopsy sampling in this study focused on a single lesion site, histopathological evaluation was restricted to that specific area, which may not fully represent the overall inflammatory status of the oral cavity.

### 4.2. Future Recommendation

Future studies should aim to evaluate the relationship between clinical findings, SDAI scores, and pretreatment biopsy results to obtain a more objective baseline representation of inflammatory severity. Relevant examinations may include inflammatory biomarkers such as IL‐6, IL‐8, and TNF‐*α*, as well as tissue immunohistochemistry. This study may serve as a foundation for larger‐scale research involving a broader range of patient characteristics to deepen our understanding of clinical and histopathological variation in FCGS. In addition, sampling multiple regions of the oral cavity should be considered to generate more comprehensive histopathological profiles and strengthen correlation analyses with clinical observations.

## Funding

This publication charge was funded by Unpad through the Indonesian Endowment Fund for Education (LPDP) on behalf of the Indonesian Ministry of Higher Education, Science and Technology and managed under the EQUITY Program (Contract No. 4303/B3/DT.03.08/2025 and 3927/UN6. RKT/HK.07.00/2025).

## Ethics Statement

This study received ethical approval from the Research Ethics Committee of Universitas Padjadjaran, as documented in Ethical Approval Letter: 692/UN6.KEP/EC/2025, dated 11 August 2025.

## Conflicts of Interest

The authors declare no conflicts of interest.

## Data Availability

The data used in this study are not publicly available due to confidentiality restrictions imposed by the participating veterinary clinic.
